# Antibiotic Types and Handling Practices in Disease Management among Pig Farms in Ashanti Region, Ghana

**DOI:** 10.1155/2014/531952

**Published:** 2014-09-11

**Authors:** John Osei Sekyere

**Affiliations:** ^1^Department of Pharmaceutics, Faculty of Pharmacy and Pharmaceutical Sciences, KNUST, Kumasi, Ghana; ^2^Department of Pharmacy, University of KwaZulu-Natal, Durban 4000, South Africa

## Abstract

Antibiotic resistance in bacteria is affected by the type of antibiotics used and how they are handled. The types of antibiotics used by 110 pig farms in the Ashanti region and the handling practices of the farmers during disease management were assessed. Injectable tetracycline, sulphadimidine, benzylpenicillin, and dihydrostreptomycin containing antibiotics were overly used by the farmers especially in the management of diarrhea, rashes, and coughs. Unsafe storage and disposal practices observed among the farms reflected the abysmal knowledge on appropriate use of antibiotics. Misdiagnosis and inadequate protection during antibiotic handling in the farms increased the risk of antibiotic resistance development and spread. The factors affecting antibiotic resistance development and spread are rife in pig farms in Ashanti region and appropriate education and veterinary interventions are needed to prevent resistant bacteria from becoming endemic in pork and pig farm communities.

## 1. Introduction

Antimicrobials are mainly used in the production of swine, cattle, and poultry and, recently, in aquaculture and crop production [1]. The antibiotics used in livestock fall into all the major classes of antibiotics used in clinical practice; there have even been cases in which antimicrobials were licensed for livestock use before their subsequent use in humans [1]. The use of antimicrobials in livestock and the husbandry practices of the farmers have been implicated as a cause of antibiotic resistance [2, 3].

Pig farmers use antibiotics for treatment, metaphylaxis, prophylaxis, and growth promotion in their farm animals [1, 2, 4]. The relatively larger farm animal populations consume more than half the antibiotics produced globally [4–6]. Diseases among pigs tend to reduce productivity by reducing feed conversion efficiency, slowing growth rate, and increasing mortalities. To safeguard their investments, farmers use sublethal doses of antibiotics to prevent diseases and promote growth [4]. To reduce the volume of antibiotics used in veterinary medicine and curtail the selection of resistant bacteria, scientists and the WHO [7] have suggested improved hygiene-based husbandry methods, veterinary supervision, and antibiotic dispensing under prescriptions only to policy makers and governments.

Because of the central role of antibiotics in disease management, a study of prevalent diseases and their management practices will aid in informing practical interventions that will aim at reducing the development and spread of antibiotic resistance as a result of antibiotic use in disease management.

Commercial pig farming in Ashanti region, Ghana, is the second most organised form of commercial animal food production after poultry in Ghana (personal communication with veterinarians, 2012). Diseases known to affect pigs include pulmonary and enteric infections, skin and organ abscesses, and mastitis [4, 5]. Swine flu and African swine fever affected several pig farms in Ghana at different periods or seasons (personal communication with veterinarians, 2012). Beta lactams, tetracyclines, aminoglycosides, sulfonamides, fluoroquinolones, polymyxins, macrolides, trimethoprim, spectinomycin, nitrofurans, and lincosamides are the antibiotics used in the treatment of pig infections globally [4, 5]; however, there is little data on the types and use of antibiotics in the pig industry in Ghana.

Antibiotics tend to break down with time. This occurs by either oxidation-reduction reactions, hydrolysis, biodegradation, or photodegradation [8, 9]. Such processes reduce the concentration of the drugs and their ability to kill the bacteria they come in contact with, allowing the exposed bacteria time to develop resistance. Environmental factors influence these processes, making storage conditions of antimicrobials very important [10].

The disposal of antibiotics with farm waste has been implicated in the spread of antibiotic resistance genes and resistant bacteria through soils, ground and surface water, atmosphere, and contaminated crops (from farm manure application or irrigation with contaminated water sources) into surrounding communities [1–3]. Consequently, describing and containing the level of such dissemination are critical to safeguarding the environment from these health hazards.

Moreover, the accumulation of antibiotics in adipose tissues [11] after their administration (antibiotic residues) has been shown to further the selection of resistant phenotypes [12, 13]. To tackle these side effects, proper antibiotic administration practices like accurate dosing, route of administration, observance of withdrawal periods, and strict adherence to manufacturers' instructions have been suggested as important solutions.

The absence of data on the type of antibiotics used and their handling practices on pig farms, especially in Ghana and Africa, is detrimental to our understanding of the factors leading to antibiotic resistance. Such studies have become important due to the public health menace facing the world as a result of bacterial resistance to antibiotics, estimated to kill 25 000 people annually in the EU alone, increasing healthcare costs worldwide [7].

As a step to encourage surveillance studies that shall help researchers track the factors influencing bacterial resistance, this study assessed the antibiotic types and their handling practices among pig farmers in disease management, allowing for recommendations to contain and prevent the development and spread of resistant bacteria through the food chain. Such awareness of the potential risks of antibiotic use in pigs on human health is essential to advise policy makers, regulatory bodies, veterinarians, and farmers on the need to address the issue of appropriate use of antibiotics in pig production.

## 2. Materials and Methods

Thirty-nine settlements within five districts in the Ashanti region of Ghana were considered for the study. 110 farms from these settlements were sampled from the list of farmers provided by the pig farmers' association in the respective districts and by the district veterinarians. The sampling was carefully done to ensure a balance between the towns and villages and farms of different financial status. These together formed an estimate of 28% of all pig farms in the region.

The study was carried out by one principal investigator and a field assistant for a period of 8 months (from May 2012 to December 2012). Scheduled regular meetings with farmers were organised during their normal farm practices such as administration of the antibiotics to the pigs. There was no major disease outbreak during these periods so histories of disease outbreaks and antibiotics used were obtained from the farmers through interviews.

Interviews were conducted with ten key informants in the pig industry on the types and proper handling practices of antibiotics. The questionnaires were in two sections: the first section had closed and open ended questions and the second section was for recording observed practices of the farmers. The observations and interviews were juxtaposed to assess the farms, using the key informers' recommendations as indicators. The questionnaires were validated on a pilot basis before the field work began.

Farms were classified as low, middle, or high income using parameters like the size of the farmland and animals, type of housing, quality of feed, number of animals sold per month, number of staff, and antibiotics used.

The questionnaires, observations, and interviews were structured into subthemes that guided the analysis. Quantitative data from questionnaires were entered into Microsoft Office Excel© software (Microsoft Corporation, version 2010, USA). Qualitative data and descriptive answers were computed in verbatim text in Microsoft Office Word© 2010 per widely accepted methods [14, 15] and were analysed manually.

The study was approved by the Kwame Nkrumah University of Science and Technology. An informed consent form which explained the purpose of the study was sent to the farm managers and district secretariats of the pig farmers' associations for approval before the interviews and observations commenced. A verbal consent was also obtained from the interviewees prior to beginning the interviews per recommended methods [14, 15].

## 3. Results

58.18% of farms (*n* = 110) were middle income, 24.55% were high income, and 17.27% were low income farms. The high income farms had more farm hands and used more antibiotics of different varieties. These farms had larger herds, between 400 and 800 herds at a time. Middle income farms formed the majority of the farms in the region, with 200 to 350 animals per year with at most two antibiotics available. Low income farms had no antibiotics at all and only bought the antibiotics when needed. They had a maximum of 100 animals per year due to financial restrictions. Low income farms were generally unhygienic due to fewer labourers; the owners either kept the farms themselves or used their family or the services of interested children.

Pig farmers resorted to antibiotics for therapeutic use only. Whereas very few farms (7/110) occasionally used clinical antibiotics, all the farms interviewed used veterinary antibiotics (Table 1). Across the districts, the tetracyclines (oxytetracycline, doxycycline, remacycline, and tetracycline) were very common (64 of 110 farms), followed by streptomycin (48/110), penicillins (48/110), and sulphadimidine (31/110). Utilisation of the fluoroquinolones, macrolides, and aminoglycosides was very minimal (Table 1). The use of heavy metal preparations like the arsenicals, copper, and zinc was not observed on any farm. Lincosamides, streptogramins, glycopeptides, *β*-lactams (except benzylpenicillin), oxazolidinones, phenicols, rifamycins, and lipopeptides were not used on any farm.

In all, the farmers used about twelve common antibiotics (Table 2). Of these, oxytetracycline had the highest number of brands and percentage usage by farmers. The number of brands was directly proportional to the popularity of the antibiotic among the farmers. Oxytetracycline, dihydrostreptomycin, procaine benzylpenicillin, and sulphadimidine together formed 82% of all antibiotics commonly used by the farmers for disease treatment.

### 3.1. Reasons and Frequency of Using Antibiotics

The farmers never used antibiotics for growth promotion and they were ignorant of it. According to the veterinarians, the use of antibiotics for growth promotion in pigs was not known and practised in Ghana. The use of antibiotics for the prevention of disease was not a common practice among the farmers. Generally antibiotics were not used frequently but only after weaning when the piglets were prone to diarrhea or in cases of disease outbreaks. Wealthy farmers used antibiotics frequently than poorer ones.

In cases of diarrhea, the farmers commonly used benzylpenicillin or benzathine penicillin, oxytetracycline, sulphadimidine, or benzylpenicillin-dihydrostreptomycin combinations. Chlortetracycline sprays were the drugs of choice for skin infections and wounds especially after castration to prevent secondary bacterial infections.

### 3.2. Knowledge Base of the Farmers Concerning Antibiotics

The farmers knew very little about antibiotics' pharmacology except for their application of knowledge from clinical antibiotics. They were educated on the uses of antibiotics by veterinarians, experienced colleagues, and veterinary shops. However, well-educated farmers followed the products legends' instructions. Knowledge of antibiotics was disseminated from experienced colleagues to inexperienced ones through the farmers' association meetings and seminars and knowledge about specific antibiotics and brands was discussed to the detriment of untried ones; most farmers within a district used a common antibiotic for all conditions.

New antibiotics were introduced to the farmers mainly by the attendants at the veterinary shops and by the veterinarians during their occasional visits or during a call at their offices. In many cases, veterinarians were not called except when there were difficulties with a strange or unmanageable disease. The farms were familiar with the brands but not the antibiotics names; it was difficult for farmers to use different brands of the same active ingredient from the same manufacturer. Due to their lower cost and better therapeutic activity, a few of the farms used powdered or granular oral antibiotics intended for poultry.

### 3.3. Off-Label Use, Withdrawal Period, and Antibiotic Residues

Very few farms (17%) knew about withdrawal periods and antibiotic residues. There were not few farms that used higher doses due to resistance to the antibiotics. Farmers commonly used antibiotics without regard to the infecting organism or the part of the body (upper respiratory or gastrointestinal) that was affected; in effect, they did not always follow what was on the labels.

### 3.4. Dosage Forms and Routes of Administration of the Antibiotics

The antibiotics dosage forms corresponded to their routes of administration (Table 3). Most of the antibiotics (>95%) were injectable liquids administered through the ear. Solid or liquid oral dosage forms, added to feed or water, happened to be designated for poultry.

### 3.5. Common Diseases and Response Practices of Farms

The common diseases affecting pigs in the region were diarrhea, worm infestations, skin rashes, and cough (Table 4). Diseases like African swine fever and swine flu were rare and occurred occasionally in few farms. Only one farm (in the Ejisu-Juaben district—Onwe town) reported on severe losses as a result of swine flu.

Diarrhea was the highest recorded diseases among the farms (Table 4) followed by skin and worms infections and anorexia, respectively. Some of the coughs observed in the pigs were idiopathic in nature and disappeared after antibiotic, multivitamin, or anthelminthic treatment. Certain worm infestations also produced coughs in the pigs. Erysipelas was not reported or seen.

Sick pigs were generally lean with poor appetites. They were inactive and prone to several secondary infections and sudden death. Farms always observed the activity and appetite of pigs and administered multivitamins and antibiotics as metaphylaxis to avoid overt disease. Disease prevalence was the highest in the Ejisu-Juaben district (Table 4).

Veterinarians were invited by the pig farmers' associations occasionally to provide lectures on the diagnosis and treatment of pig diseases. Many farmers learnt to treat their own animals or consulted their more experienced colleagues to avoid charges involved in engaging the services of a veterinarian. Moreover, the small number of veterinarians (an average of one per district) also made it difficult for all the farmers to get their services at a given time or for the veterinarians to go on regular farm visits. The veterinarians, on the other hand, also complained of inadequate resources to help them visit the distant farms regularly.

### 3.6. Diagnosis

The farms did not know the name of the diseases that affected their animals but their symptoms; consequently, the conditions herein stated were based primarily on symptoms and farmers' descriptions. They therefore treated the symptoms instead of the underlying etiology. Where a veterinarian's intervention in difficult-to-treat disease became successful, the farms learned from it and carefully followed the same protocol in similar future occurrences.

### 3.7. Storage and Disposal of Antibiotics

37.18% of the farms stored their antibiotics on the bare floor, 32% on wooden shelves, 12.82% in card boxes kept on floors, 12.82% in polythene bags (hanged on walls), and 5.13% in cupboards. These storage sites were not securely locked and could easily be accessed by unauthorised persons and children.

Pig farmers disposed of their used antibiotic containers and wastes mainly by throwing them into drains, onto refuse dumps, or on the bare ground (Table 5) instead of burying them as recommended. In the Ejisu-Juaben district farmers dumped most of their antibiotic wastes on the bare ground around the farms whereas, in Atwima Nwabiagya, the proportion of antibiotics disposed through the sewage was very close to that disposed onto the soil. Most farmers in Bosomtwe and Atwima Kwanwoma and Kwabre East districts disposed of their medicines into drains.

### 3.8. Protective Gear Worn by Farmers

Most farms (68%) used some protective clothing whenever they administered antibiotics to their animals (Table 6). All respondents in Bosomtwe and Atwima Kwanwoma districts used some form of protection. Rubber boots and gloves were the major items worn by the farmers. The proportion of farmers using protective outfit on all their body parts reduced as the number of protective items used increased. After handling antibiotics, workers on 96% of the farms washed their hands. Of this number, about 83% washed their hands using soap and water, 9% used water only, and another 8% used disinfectants with water. Tools used for handling and administration of antibiotics were mostly washed with soap and water (83%), with the remaining farms using water only (9%) or other disinfectants (8%).

## 4. Discussion

The types and handling practices of antibiotics used in both clinical and veterinary medicine have been implicated in the development and spread of resistant bacterial phenotypes that is affecting the therapeutic efficacy of current antibiotics and food security [1, 2, 7]. This study aimed at assessing the practices and types of antibiotics prevalent on Ghanaian pig farms, using the Ashanti region as a case study. The types, frequency, and user practices of the antibiotics were affected by the financial status of the farms. Due to the larger number of animals and financial ability of middle income and high income farms, they used more antibiotics than lower income farms. The smaller numbers and poor finances of low income farms made it economically untenable to use more antibiotics. Consequently, more variety and quantity of antibiotics were used on medium and larger farms than smaller ones. As a result, the prospects of resistance to various antibiotics in bacteria in the farm environment and in the pigs are higher in middle and higher income farms than in smaller income farms. Subsequently, increased veterinary attention and education should be directed more towards middle and higher income farms than the smaller income ones to minimise practices that will increase antibiotic abuse and resistance.

Antibiotics used for growth promotion and prophylaxis were largely unknown among the farms. This is better than that reported in Kenya [16, 17] and a welcoming observation as the use of antibiotics for prevention and growth promotion is known to select for resistant bacterial phenotypes [1, 2]. Moreover, the use of clinical antibiotics (Table 1) in disease management was rare. The use of antibiotics intended for clinical use is advised against as a cause of the spread of antibiotic resistance from farms to communities and treatment centres [3, 7]. Hence, the use of mainly veterinary antibiotics for basically therapeutic purposes among the farms reduces the risks of antibiotics resistance development and spread.

Nevertheless, the farms mainly depended on the tetracyclines (oxytetracycline, doxycycline, remacycline, and chlortetracycline), sulphadimidine, dihydrostreptomycin, and benzylpenicillin for the management of most diseases. And these or other antibiotics of the same family are used extensively in managing clinical bacterial infections in Ghana. Consequently, the possibility of cross- and coresistance [1–3, 7] makes their broad use among the farms unsafe in terms of food security and public health as bacteria with resistance to these antibiotics could affect consumers [3, 5]. For instance, doxycycline, streptomycin, and injectable benzylpenicillin are used in the treatment of gonorrhea, otitis, oral infections, and so forth (personal observation in hospitals and pharmacies), and consumption of pork or contamination from farmers with resistant bacteria could make these antibiotics useless. On the other hand, the relatively lower use of the fluoroquinolones, macrolides, and gentamicin among the farms is relieving to some extent because ciprofloxacin is the main antibiotic of choice in treating cholera and several resistant bacterial infections in Ghana (personal observation in hospitals and pharmacies). Subsequently, there is the need to educate farmers to shift from these antibiotics to phenicols, lincosamides, and glycopeptides as these classes of antibiotics are not common in clinical use in Ghana.

The knowledge of the farmers concerning antibiotics, withdrawal periods, and dosages was found to be very low. Moreover, the farmers depended more on fellow farmers than veterinarians for antibiotic knowledge, which resulted in the use of the same antibiotics and similar handling practices among farms in close proximity or within the same district. Poor dosing practices, for example, were common when an antibiotic failed to treat an infection. In measuring out the antibiotics, the farmers lacked adequate measuring instruments to mete out correct dosages. Different antibiotics were then tried and also abused till the disease was treated. Because the farmers lacked adequate knowledge in antibiotics, they hardly knew about the similarity of different antibiotic brands with the same active ingredients; thus, different antibiotic brands of the same active ingredient would be used without improvement in the disease condition. Consequently, errors in antibiotic handling and administration were common among the farms. Nonadherence to dosing and withdrawal period was reported by Addah et al. [18] among several livestock farmers in Northern Ghana, practices that increase antibiotic residues in animal foods [19]. The number of farmers (17%) who knew about antibiotic residues and the withdrawal period is concerning. The presence of antibiotics and resistant bacteria in pork and other animal products results when there is off-label use [2, 3, 5, 7, 19]. Antibiotic and resistant bacteria contaminated pork are known to threaten the health of consumers [5].

Most (>95) antibiotics used in the pig farms were liquid intravenous (IV) injections administered through the veins in the ear. The farms administered the antibiotics per animal when there was a disease condition and not to all animals through the feed or water. The IV route, however, is not without risks as the antibiotics easily settle in the adipose tissues over a longer period of time, putting consumers of pork fat at high risk of antibiotic exposure. The major advantage of not administering the antibiotics through feed or water, as is the case in poultry [4], is that the unnecessary exposure of healthy animals to antibiotics was avoided. Consequently, the risk of selecting resistant commensals that will later be shed into the environment through the faeces was limited.

The study showed high prevalence of diarrhea, worm infestations, cough, and skin infections. Subsequently, antibiotics used on the farms were targeted towards alleviating these diseases. From the observations made, these diseases could be easily controlled by adopting improved hygienic practices on the farms together with increased use of probiotics. The inadequate knowledge of the farmers in animal diseases made them depend on symptomatic treatment instead of targeting the etiological agent. As a result, antibiotics were tried without proper diagnosis. Several disease conditions result in diarrhea-like symptoms. Many of the diarrheal cases could be due to coccidiosis though none of the farmers used coccidiostats; consequently, the failure of antibiotic therapy in the farms may well be due to misdiagnosis. Ironically, however, farmers blamed the failures on the brands or dosage and tend to revert to other brands or increased dosage. Bacterial toxins in the GIT cause diarrhea [20]—using antibiotics will kill the bacteria but not the toxins and coadministration with toxin binders may salvage the diarrhea, but this knowledge was unknown in the farms. Similarly, not all coughs may be bacteria related. Further investigations into the causes of the coughs could reduce the administration of antibiotics for all coughs.

Rashes on pigs had several etiologies. They could be secondary bacterial or fungal infections from bruises obtained in a fight, scratches from a rough sty wall, or ectoparasite infestations. Ectoparasites made the animals restive and itchy, forcing them to scratch their hides on the pen walls. Thus, the skin rashes led to a vicious cycle of infections and reinfections that affected the hides and health of the animals. The diseases prevalent on the farms visited had little similarities with that determined by Perry and colleagues [21] in Africa. The only disease within Perry's ranking that agreed with this study was ectoparasites and helminthosis. Diarrhea was not ranked among the top diseases. African swine fever, which was the highest pig disease in Africa [21], was not prevalent or endemic in the Ashanti region during the study period.

The storage conditions of antibiotics in the farms were suboptimal because the storage environments of the antibiotics were prone to temperature fluctuations which hastens antibiotic decomposition, reducing its concentrations and efficacy [22], thus promoting resistance in exposed intestinal bacteria [3, 5, 23, 24]. The underperformance of some of the antibiotics as reported by the farmers could be due to their antibiotic storage sites. The dumping sites of antibiotic wastes, used antibiotic containers, and pig faeces (Table 5) inevitably introduce antibiotics, resistance genes, and resistant bacteria into the farm environment, especially, when resistant enteric bacteria have been shown to possess the ability of transferring their resistant genes to soil bacteria [25]. It could also further pollute surface and ground water with antibiotics, resistance genes, and resistant bacteria [2]. Furthermore, the antibiotics could easily be accessed and abused in these storages sites, as drugs intended for use in farm animals may be used in humans voluntarily or involuntarily [26].

The protective outfits used by the farmers were in some of the cases not adequate, increasing the risk of farmers being exposed to antibiotics. As farmers mostly used soap and water to wash (disinfectants were used on less than 10% of the farms) the possibility of being contaminated with antibiotics is very great. Such exposures allow for the development of resistance in skin and nasal bacterial flora as reported by Levy [24]. Soap and water are not effective agents in annihilating skin and nasal bacteria. Thus the farmers could serve as conduits to transfer resistant bacteria into the communities [23, 24, 27–30]. Further studies to evaluate the resistance of bacteria from the skin of farmers will be important to assess the risks and levels of resistant bacteria among the farmers.

## 5. Conclusion

Inadequate knowledge about antibiotics and their effect on the environment and public health among pig farmers made them use and dispose of antibiotics imprudently, increasing the risk of antibiotic resistance development and spread. Education on the importance of pro- and prebiotics, improved farm hygienic practices, strict laws regarding the sales, and use of antibiotics and increased veterinarian interventions on the farms are important to limit the occurrence of diseases and subsequent use of antibiotics.

## Figures and Tables

**Table 1:** Types of antibiotics used by pig farmers in selected districts in the Ashanti region of Ghana.

Antibiotics	Ejisu-Juaben (*n* = 43)	Bosomtwe/Atwima	Atwima	Kwabre East (*n* = 21)
		Kwanwoma (*n* = 24)	Nwabiagya (*n* = 20)	
Penicillin-streptomycin	25	3	10	10
Tetracyclines	31	9	16	8
Tylosin	4	0	2	0
Enrofloxacin (+norfloxacin)	3	0	2	5
Sulfadimidine	9	2	13	7
Amoxicillin	1	0	1	0
Metronidazole (clinical)	6	0	0	0
Erythromycin	1	0	0	4
Trimethoprim	2	0	2	0
Gentamycin	2	0	2	0

**Table 2:** Active ingredients and brands of antibiotics used by pig farmers.

Active ingredient of antibiotics	Number of brands	Number of user farms
Procaine/benzathine benzylpenicillin	6	45
Dihydrostreptomycin	8	50
Chlortetracycline	1	9
Gentamicin	1	4
Sulphadimidine	8	33
Trimethoprim	1	4
Oxytetracycline	21	58
Enrofloxacin	4	9
Erythromycin	2	5
Norfloxacin	1	2
Amoxicillin	1	1

**Table 3:** Dosage forms and routes of administration of antibiotics per district.

District	Dosage form	Oral route	Intravenous route	Surface/topical route	Total
Ejisu-Juaben (*n* = 43)	Solid/powder	17	0	0	17
	Liquid	6	57	0	63
	Aerosol	0	0	2	2
	Total	**23**	**57**	**2**	**82**

Atwima-Nwabiagya (*n* = 20)	Solid/powder	1	0	0	1
	Liquid	4	37	0	41
	Aerosol	0	0	6	6
	Total	**5**	**37**	**6**	**48**

Bosomtwe and Atwima Kwanwoma	Solid/powder	0	0	0	0
	Liquid	0	13	0	13
	Aerosol	0	0	1	1
	Total	**0**	**13**	**1**	**14**

Kwabre East	Solid/powder	11	0	0	11
	Liquid	3	20	0	23
	Aerosol	0	0	0	0
	Total	14	20	0	34

Grand total		42	127	9	178

**Table 4:** Common diseases on pig farms in different districts.

Disease	Ejisu-Juaben district (*n* = 32)	Bosomtwe and Atwima Kwanwoma (*n* = 6)	Kwabre East (*n* = 11)	Atwima Nwabiagya (*n* = 20)	Total (*n* = 96)
Skin rashes	11	1	3	4	19
Cough	5	0	0	0	5
Diarrhea	30	6	7	8	51
Worms infestations	6	0	0	2	8
Anorexia	3	0	1	9	13
Pneumonia	2	0	0	0	2

**Table 5:** Disposal sites of antibiotic waste and containers.

Disposal sites of antibiotics	Ejisu-Juaben (*n* = 43)	Atwima Nwabiagya (*n* = 20)	Bosomtwe/Atwima Kwanwoma (*n* = 24)	Kwabre East (21)
Refuse dump	0	6	4	4
Sewage/drains	23	17	10	16
On bare ground/soil	38	19	0	5

**Table 6:** Types of protective clothing used by farms.

Protectives used	Ejisu-Juaben (*n* = 29)	Bosomtwe/Atwima Kwanwoma (*n* = 4)	Atwima Nwabiagya (*n* = 12)	Kwabre East (*n* = 8)
Rubber boots	18 (62%)	3 (75%)	5 (41.7%)	5 (62.5%)
Gloves	15 (51.7%)	1 (25%)	7 (58.3%)	6 (75%)
Masks	3 (10.3%)	0	0	4 (50%)
Glasses	1 (3.4%)	1 (25%)	0	0
Overcoat	15 (51.7%)	2 (50%)	1 (8.3%)	5 (25%)
